# The African Polio Laboratory Network: Advancing Regional Poliovirus Detection, Genomic Surveillance, and Eradication

**DOI:** 10.3390/vaccines14090766

**Published:** 2026-09-02

**Authors:** Brook Tesfaye, Terna Nomhwange, Shelina Moonsamy, Julius E. Chia, Maryceline M. Baba, Adedayo Omotayo Fanaye, Mesfin Tefera, Yogolelo Riziki, Peter Borus, Josephine Bwogi, Marie-Claire Endegue, Mariette Glitho, Idah Ndumba, Priscilla Mosoke, Ousmane M. Diop, John Kofi Odoom, Martin Faye, Gedi Mohamed, Anfumbom Kfutwah

**Affiliations:** 1 Polio Eradication Program, Office of the Regional Director, World Health Organization, Regional Office for Africa, Brazzaville P.O. Box 06, Congo; nomhwanget@who.int (T.N.); jchia@who.int (J.E.C.); glithoe@who.int (M.G.); ndumbaid@who.int (I.N.); priscillab@who.int (P.M.); mohamedge@who.int (G.M.); kfutwaha@who.int (A.K.); 2 Centre for Vaccines and Immunology, National Institute for Communicable Diseases, a Division of the National Health Laboratory Service, Johannesburg 2192, South Africa; shelinam@nicd.ac.za; 3 Department of Medical Laboratory Science, College of Medical Sciences, University of Maiduguri, Maiduguri 600004, Nigeria; marycelinb@yahoo.com; 4 Department of Virology, College of Medicine, Faculty of Clinical Sciences, University Ibadan, Ibadan 200005, Nigeria; adedayoraji@yahoo.com; 5 Ethiopian Public Health Institute, Addis Ababa 1000, Ethiopia; mesfinteffera@gmail.com; 6 Institute National de Recherche Biomédicale, Kinshasa 5345, Democratic Republic of the Congo; yogolelori@gmail.com; 7 World Health Organization, Nairobi 00100, Kenya; borusp@who.int; 8 EPI Laboratory, Uganda Virus Research Institute, Entebbe P.O. Box 49, Uganda; jbwogi@uvri.go.ug; 9 Service de Virologie, Centre Pasteur du Cameroun, Yaoundé 1274, Cameroon; endegue@pasteur-yaounde.org; 10 World Health Organization, 1211 Geneva, Switzerland; diopo@who.int; 11 Virology Department, Noguchi Memorial Institute for Medical Research, College of Health Sciences, University of Ghana, Accra 00233, Ghana; jodoom@noguchi.ug.edu.gh; 12 Institute Pasteur, Dakar 12900, Senegal; martin.faye@pasteur.sn

**Keywords:** Africa polio laboratory network, vaccines, polio eradication, circulating vaccine-derived poliovirus (cVDPV), genomic surveillance, molecular epidemiology, laboratory quality management

## Abstract

Background: Sustaining progress toward polio eradication requires not only effective vaccination but also a resilient laboratory network capable of timely poliovirus detection, confirmation, and genetic characterization. In the WHO African region, the African polio laboratory network provides essential virological support for eradication efforts; however, persistent circulating Vaccine-derived Polioviruses (cVDPVs) outbreaks and increasing demands of genomic surveillance continue to challenge laboratory capacity. Despite its critical role, evidence describing the operational performance, adaptive capacity, and evolution of regional polio laboratory network remains limited. This study evaluates the performance and operational experience of the African polio laboratory network in 2025, highlighting contributions to poliovirus detection, genomic surveillance, quality assurance, and outbreak response, while sharing lessons to inform regional public health systems. Methods: A retrospective secondary data analysis was conducted using laboratory surveillance and outbreak databases, supplemented by reviews of programmatic reports. Data were cleaned and harmonized prior to analysis. Descriptive analyses were performed to assess laboratory workload, timeliness indicators, genomic capacity, quality assurance performance, and operational challenges. Qualitative review of programmatic documents was conducted to identify contextual factors influencing performance. Results: In 2025, 77,230 Acute Flaccid Paralysis (AFP) specimens were analyzed across 16 laboratories. Ninety-two percent of specimens met the timeliness target for virus isolation, and 94% met the target for PCR-ITD. Genomic sequencing confirmed 20 cVDPV outbreaks in 10 countries. The establishment of new sequencing laboratories in Uganda and Nigeria reduced turnaround times, and a 2025 expansion plan aimed to build sequencing capacity in 12 additional countries. All laboratories assessed in 2024 for virus isolation, PCR-ITD, and environmental surveillance exceeded quality assurance accreditation thresholds and were rated “Pass”. The pilot implementation of Direct Detection Nanopore Sequencing (DDNS) techniques is expected to further strengthen genomic surveillance. Challenges remain, including stock out of essential supplies, uneven workloads, and infrastructure gaps. Conclusions: The African polio laboratory network has demonstrated sustained capacity, adaptability, and innovation in supporting polio eradication activities in the WHO African region. This study provides evidence on the importance of strengthening laboratory systems, expanding genomic capacity, and maintaining quality assurance mechanisms to support the final phase of polio eradication and broader public health surveillance.

## 1. Introduction

The success of any disease eradication initiative depends not only on vaccines and immunization strategies but also on a robust laboratory network that generates timely and actionable data [1,2]. Public health laboratories are fundamental to effective surveillance systems, enabling rapid detection, outbreak confirmation, and pathogen characterization to inform response strategies [1,3]. In the global effort to eradicate poliovirus, laboratory networks have played a pivotal role in translating diagnostic evidence into coordinated action at national, regional and global levels [4,5].

Polio is a highly infectious viral disease that can cause irreversible paralysis, leading to death in rare cases [6,7]. Poliovirus is a positive-sense single stranded ribonucleic acid (RNA) virus belonging to the genus Enterovirus within the family Picornaviridae. Its genome is approximately 7.5 kb in length and comprises a single open reading frame flanked by 5′ and 3′ untranslated regions. The viral capsid consists of four structural proteins (VP1–VP4), with the VP1 region serving as the primary target for molecular characterization because it contains the major antigenic sites and accumulates nucleotide substitutions that enable differentiation of poliovirus serotypes, vaccine-related strains, vaccine-derived polioviruses (VDPVs), and Wild Polioviruses (WPV) [8,9].

The use of oral polio vaccine (OPV) and inactivated polio vaccine (IPV) in routine immunization, alongside intensified surveillance systems, has driven substantial progress toward global eradication [7,10]. A major milestone was reached in August 2020, when the Africa Regional Certification Commission (ARCC) declared the African Region free of indigenous wild poliovirus (WPV) [11]. However, this achievement remains fragile, as ongoing outbreaks of circulating vaccine-derived polioviruses (cVDPVs) continue to pose a substantial threat to regional eradication goals [12,13].

Outbreaks of cVDPVs reveal gaps in surveillance systems and underscore the need for robust laboratory infrastructure capable of timely detection and characterization of pathogens while guiding outbreak response activities [9,14,15]. Central to this effort is the Global Polio Laboratory Network (GPLN), the largest public health laboratory network ever established, comprising more than 140 laboratories in 92 countries, operating through a three-tier system [2,14]. The first tier is National Polio Laboratories (NPLs) which serves the host country and in some cases neighboring countries, conducting virus isolation and Intratypic Differentiation (ITD) by Polymerase Chain Reaction (PCR). Recently sequencing activities have been implemented to the routine activities of some NPLs. The second tier, Regional Reference Laboratories (RRLs), performs referral testing in the region and sequencing in addition to NPLs functions. The third tier, Global Specialized Laboratories (GSLs), carries out all activities of NPLs and RRLs and is responsible for preparation and distribution of reagents, and Proficiency Test (PT) panels for annual quality assurance and quality control [14]. Collectively, the GPLN supports the eradication initiative by providing virological confirmation and genetic sequencing of polioviruses to guide timely interventions [5,9,14].

**Figure 1:**
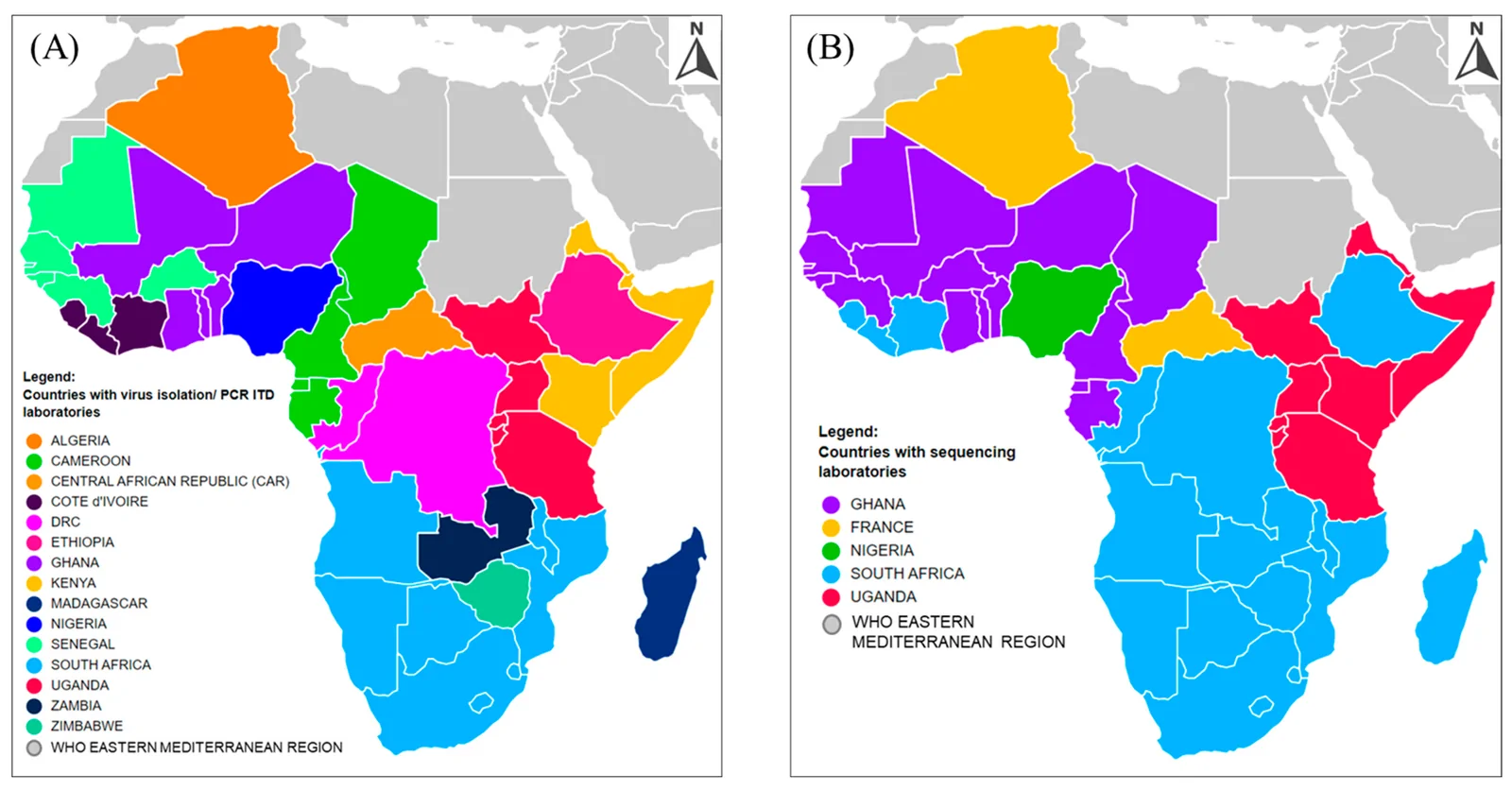
Geographic distribution and service coverage of the African polio laboratory network, 2025. Map illustrating countries with operational polio laboratories and their respective referral catchment areas for specimen testing. Countries are color-coded to indicate laboratory referral linkages and service coverages, with each color representing countries served by the same reference laboratory. Countries hosting laboratories are indicated by dots. (**A**) Primary testing network: virus isolation and intratypic differentiation (PCR-ITD). (**B**) Secondary testing network: VP1 sequencing. Poliovirus isolates from the Central African Republic (CAR) and Algeria are sequenced outside the African region, in France. Nigeria hosts two polio laboratories. Although Somalia and Djibouti are part of the WHO Eastern Mediterranean Region (EMRO), their specimens are processed within the African laboratory network.

The African polio laboratory network functions as a key component of the GPLN, supporting poliovirus detection and outbreak management in the World Health Organization (WHO) African region [2,14,15]. As indicated in Figure 1, the network includes 16 WHO-accredited polio laboratories across 15 countries, with two laboratories in Nigeria, forming a geographically distributed and interconnected network [2]. These laboratories conduct virus isolation and PCR-ITD on samples from AFP and environmental surveillance samples [5,14,15]. To ensure compliance with international quality standards, they undergo annual accreditation by WHO-GPLN reviewers [14].

Genomic sequencing has become an essential tool for tracking poliovirus transmission, identifying transmission chains, monitoring viral evolution, and informing Supplementary Immunization Activities (SIAs) [9,16,17,18]. Following virus isolation and PCR-ITD, viral RNA is extracted from poliovirus isolates and the complete VP1 coding region is amplified using standardized RT-PCT protocols. The amplified VP1 products are then sequenced using Sanger sequencing platforms, and the resulting nucleotide sequences are analyzed to determine poliovirus serotypes, identify VDPVs, estimate genetic divergence from Sabin vaccine strains, and establish phylogenetic relationships for molecular epidemiological investigations.

Despite these advances, persistent cVDPV outbreaks and, in some cases, delays in genomic sequencing have highlighted gaps in genomic surveillance that hinder outbreak management and vaccination planning [9,15]. Until beginning of 2025, only the polio laboratories in South Africa and Ghana had Poliovirus sequencing capacity. The establishment of sequencing laboratories in Uganda and Ibadan, Nigeria, in the beginning of 2025 aimed to reduce genetic sequencing Turnaround Time (TAT), alleviate workload, and strengthen regional capacity. Continued investments in workforce training, bioinformatics, and diagnostic infrastructure are also enhancing the network’s ability to support genomic surveillance [14,15].

The eradication of all WPVs in the WHO Africa region was facilitated by the strength of the laboratory network [9,14]. As the region shifts focus to sustaining eradication gains and interrupting cVDPVs outbreaks, there is a strategic imperative to expand and integrate genomic capacity across the network to ensure timely confirmation and response, not only for polioviruses but also for other infectious diseases [9,14].

Our paper provides insights into the contributions of the GPLN and more specifically the African polio laboratory network to the polio eradication goals in the African region. It describes the structure, capacity, and performance of the network in 2025 and evaluates key indicators, including TAT, sequencing coverage, and quality assurance including accreditation outcomes [19].

## 2. Methods

### 2.1. Study Area

This study focuses on the polio eradication program in the WHO African region, one of six WHO regions globally, comprising 47 member states and a population exceeding one billion [20]. The region bears a disproportionate burden of infectious diseases and has played a pivotal role in global public health initiatives, including the Global Polio Eradication Initiative (GPEI) [21,22].

### 2.2. Study Design and Period

Herein, we evaluated laboratory performance indicators through a retrospective secondary data analysis of AFP stool specimens processed in WHO-accredited polio laboratories across the WHO African region in 2025. In addition, document reviews were conducted to assess programmatic and technical reports, including WHO surveillance bulletins, laboratory network performance reports, and accreditation mission outcomes.

These methods enabled the assessment of both quantitative performance indicators and the qualitative contributions of the African polio laboratory network to the global eradication efforts. In addition, systematic evaluations of all data sources were performed to ensure consistency and completeness.

### 2.3. Data Collection

Our study utilized multiple data sources generated by the polio eradication program in the WHO African region. The primary source was the polio laboratory database, which contains standardized AFP cases specimen-level data and reported poliovirus isolates used to support surveillance, monitoring, and programmatic response activities [23].

Additional data were obtained from epidemiological bulletins and technical reports from 2025 laboratory quality management and accreditation missions, accessed via the program portal. Workshop proceedings organized by the Africa polio laboratory network were also reviewed to provide contextual insights.

### 2.4. Laboratory Key Performance Indicators

In line with the Global Polio Surveillance Action Plan (GPSAP) 2025–2026 strategic plan, our study used four standard key performance indicators [23]. The indicators, along with their definitions and targets, are outlined below:

Workload analysis: The number of stool samples analyzed by polio laboratories.

Timeliness of virus isolation results: Proportion of specimens with virus isolation results within 14 days of receipt of AFP specimen at the WHO-accredited laboratory (target ≥ 80%).

Timeliness of ITD results: Proportion of specimens with ITD results within 7 days of virus isolation results (target ≥ 80%).

Timeliness of sequencing results: Proportion of specimens with final sequencing results within 7 days of arrival at the sequencing laboratory (target ≥ 80%).

### 2.5. Laboratory Quality Management

To ensure sustained quality and compliance with global standards, all laboratories within the GPLN participate in a structured two-year accreditation cycle. Accreditation follows WHO protocols and includes GPLN standardized checklists, performance evaluations, and review of laboratory key activities [5]. Annual refresher training is also conducted for laboratory personnel, focusing on quality management and successes in Proficiency tests (PT) for all recommended poliovirus detection methods [24]. Laboratories are further required to perform in-house validation of accepted methods through an approved procedures to ensure consistency and technical reliability [5,24]. These activities are integral to the GPLN’s broader quality assurance framework and are critical for maintaining laboratory readiness, supporting accurate diagnostics, and enabling timely public health responses within the polio eradication program [5,14,24].

### 2.6. Data Collection Tools, Analysis and Presentation

Prior to analysis, the laboratory database underwent comprehensive data cleaning and data transformation to ensure accuracy and consistency. Records with missing or incomplete values for key performance indicators were excluded. This approach aligns with best practices in polio data management, where standardized tools, validation rules, and routine data quality checks enhance the reliability of surveillance data [25,26].

Data analysis combined descriptive statistics with qualitative content analysis. Descriptive statistical methods, including frequencies, percentages, medians, and ranges were used to summarize laboratory network performance indicators over time and across laboratories. No inferential statistical tests were performed, as the study was based on routine surveillance and laboratory program monitoring data and was intended to provide a descriptive assessment of network performance rather than test statistical hypothesis. Content analysis of qualitative sources such as workshop proceedings and accreditation reports identified recurring themes and contextual insights. These methods support the generation of meaningful outputs and feedback, ultimately strengthening the quality and utility of polio surveillance data [25,27]. Data management, transformation and analyses were performed using Microsoft Excel (Version 2016), Python programming (version 3.12), and R programming (Version 4.3.2) with various analytical libraries. Results are presented using descriptive tables, graphical visualization, and geographic maps charts, tables, and maps to illustrate key findings.

### 2.7. Ethical Considerations

All personalized and identifiable information were removed from the data sources prior to analysis to ensure confidentiality.

## 3. Results

### 3.1. Workload Analysis and Timeliness of Sample Processing

**Figure 2:**
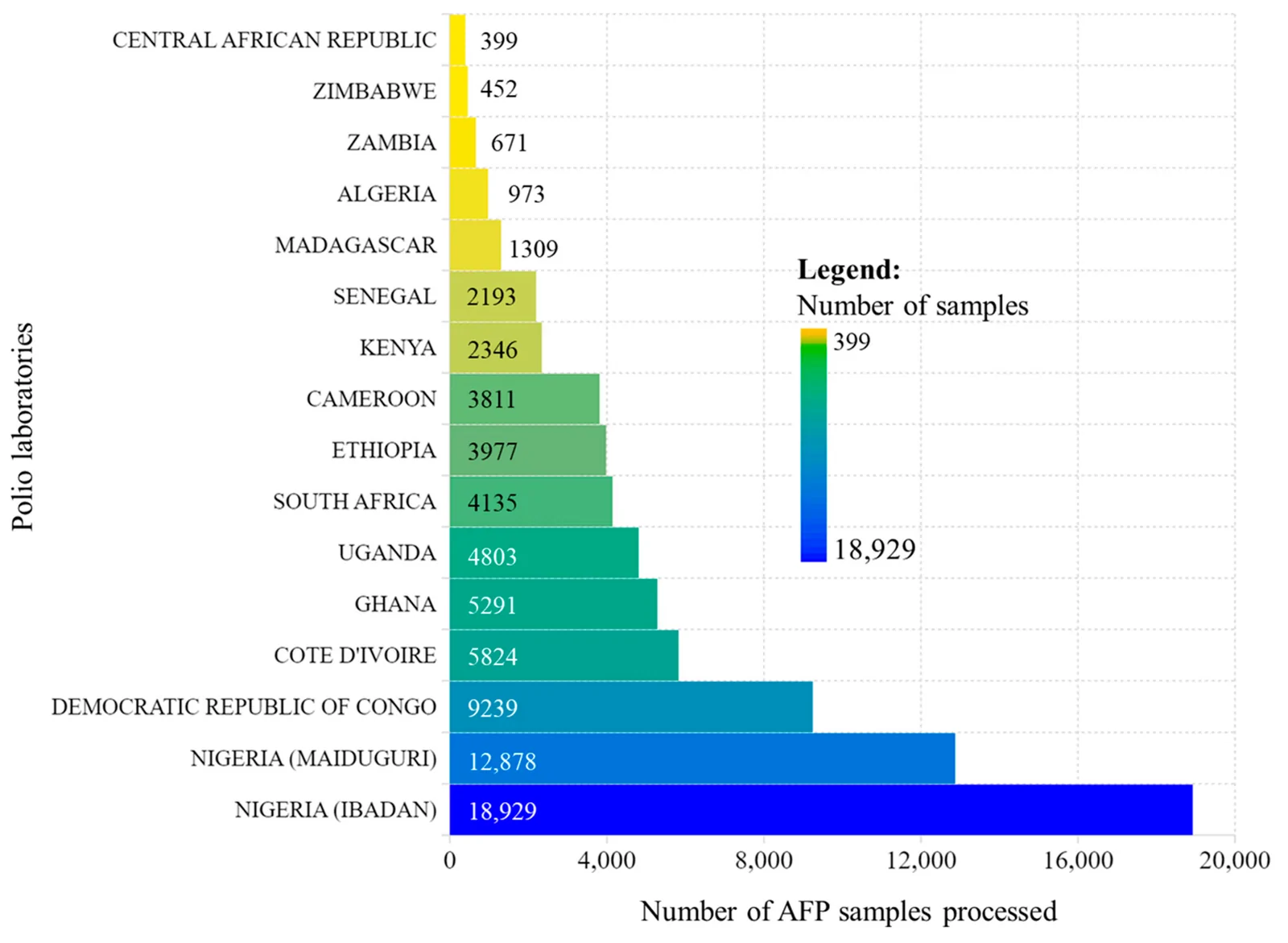
Number of AFP stool specimens analyzed by WHO-accredited polio laboratories in the WHO African region, 2025. Bar lengths indicate total workload volume, and the color gradient represents relative workload intensity.

In 2025, the Africa polio laboratory network analyzed 77,230 AFP stool specimens (Figure 2). Among individual laboratories, Ibadan (Nigeria) processed the highest number of specimens (18,929), followed by the Maiduguri (Nigeria) laboratory with 12,878 specimens, and the Democratic Republic of Congo (DRC) laboratory with 9239 specimens. Laboratories in Nigeria and DRC collectively analyzed 53% of the total regional samples, underscoring their critical contribution to poliovirus diagnosis in the region. Mid-tier laboratories in Cote d’Ivoire (5824), Ghana (5291), and Uganda (4803) also made substantial contributions. In contrast, laboratories in Zimbabwe and the CAR processed the least specimens, totaling 452 and 399, respectively.

### 3.2. Turnaround Time Performance for Poliovirus Isolation

**Figure 3:**
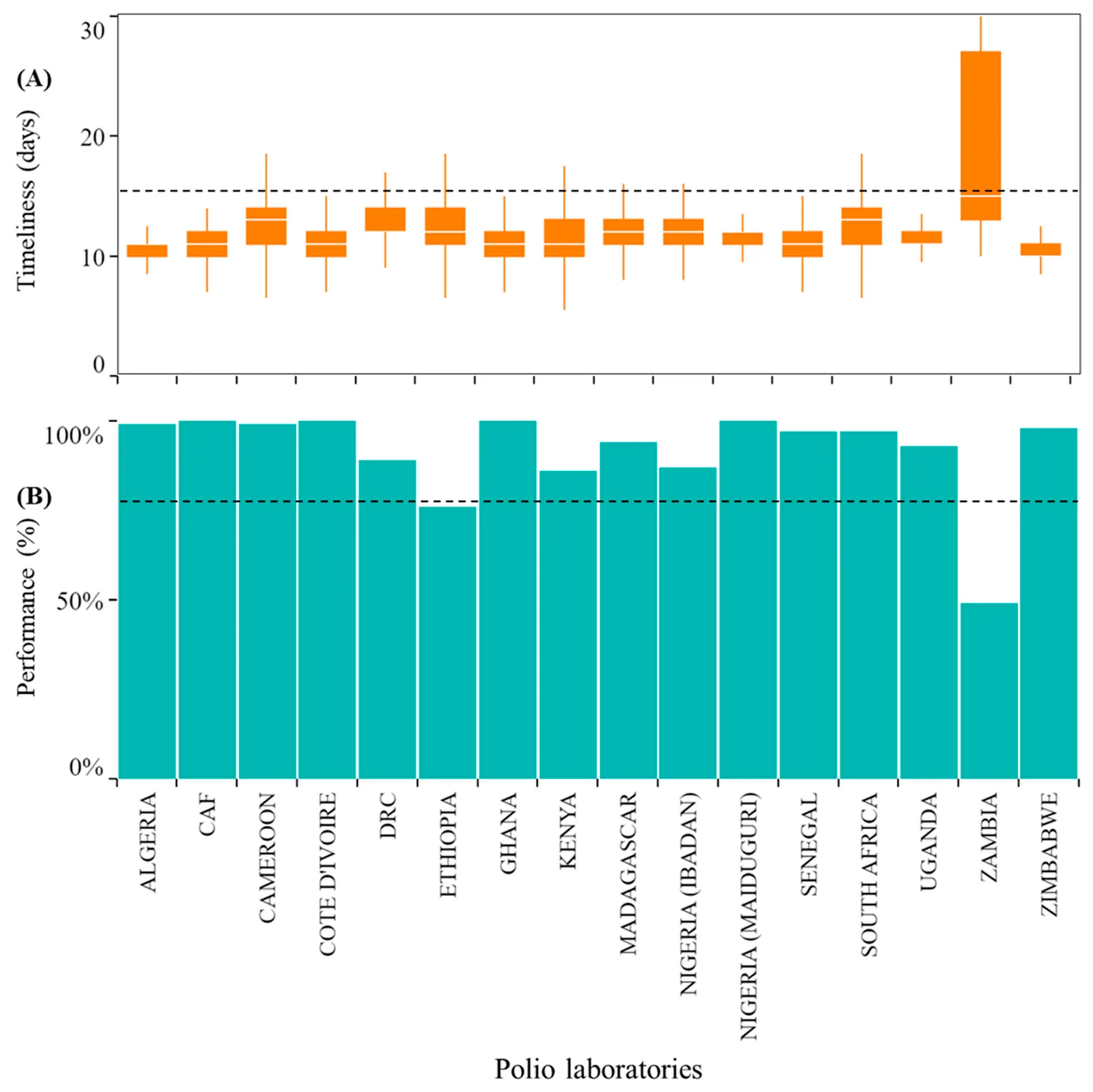
Turnaround time performance for poliovirus isolation, WHO African region, 2025. (**A**) Boxplots showing the distribution of turnaround times (days) from sample receipt to reporting of virus isolation results across laboratories. (**B**) Proportion of samples with final poliovirus isolation results within 14 days of arrival at the polio laboratory. The dashed lines indicate the WHO-recommended targets: <14 days for turnaround time and >80% of samples meeting the timeliness threshold.

The TAT for poliovirus isolation and PCR-ITD differed among the African polio laboratories. For virus isolation (Figure 3B), all laboratories except Ethiopia and Zambia achieved the WHO-recommended target of 80%. Figure 3A shows the distribution of TATs across the laboratories. Median TATs ranged from 10 to 15 days, with most laboratories demonstrating relatively narrow interquartile ranges (IQRs). Zimbabwe demonstrated the shortest median TAT (10 days; IQR: 10–11, whereas, Zambia had the longest and most variable TAT (15 days; IQR: 13–27). Median TATs of 11 days were observed in Algeria (IQR: 10–11), CAR (IQR: 10–12), Cote d’Ivoire (IQR: 10–12), Ghana (IQR: 10–12), Kenya (IQR: 10–13), Senegal (IQR: 10–12), and Uganda (IQR: 11–12); 12 days in Ethiopia (IQR: 11–13), Ibadan (Nigeria) (IQR: 11–13), Madagascar (IQR: 11–13), Maiduguri (Nigeria) (IQR: 11–12), and DRC (IQR: 12–14); and 13 days in Cameroon (IQR: 11–14) and South Africa (IQR: 11–14).

### 3.3. Turnaround Time Performance for PCR-ITD

**Figure 4:**
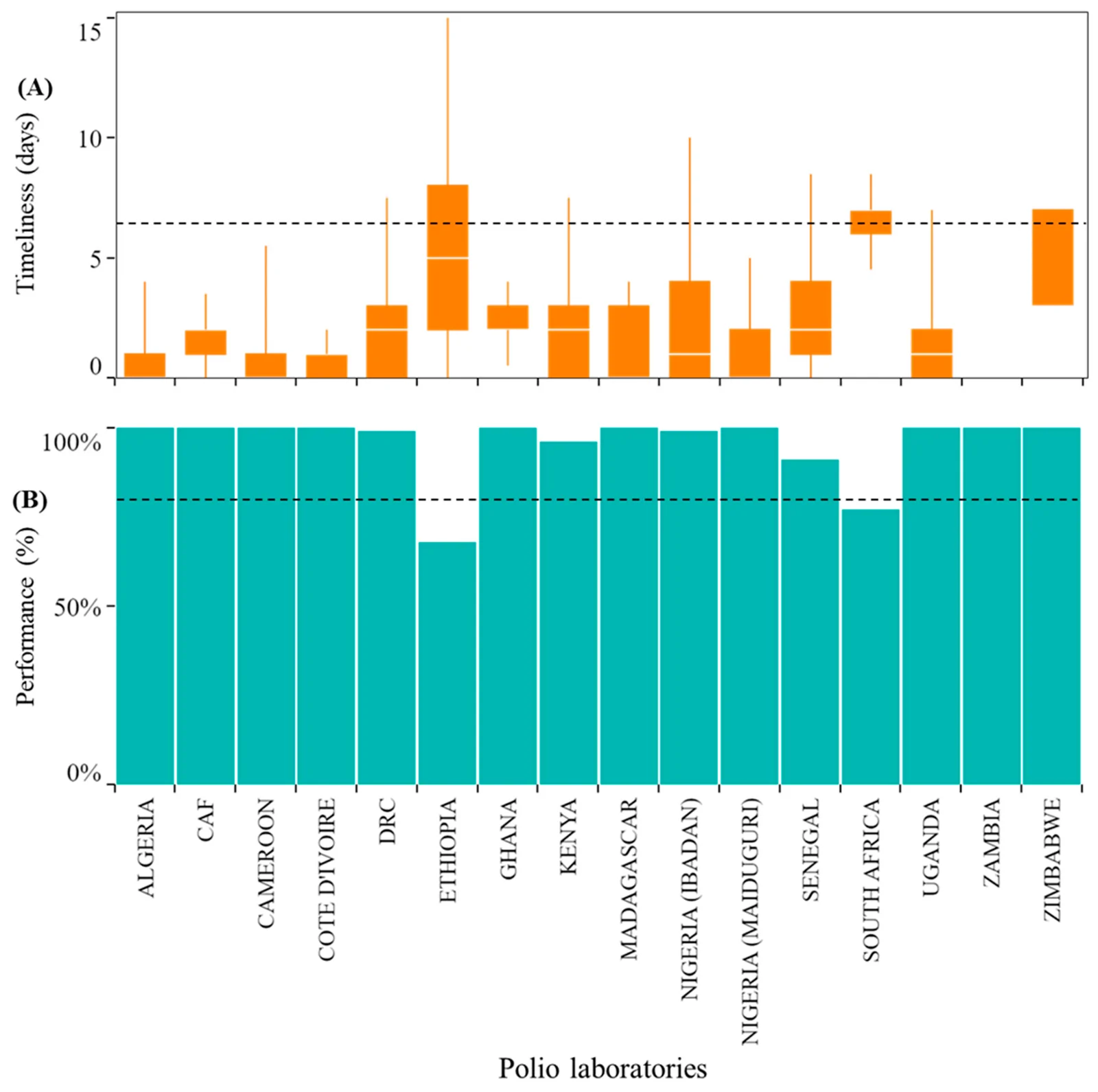
Turnaround time performance for PCR-ITD, WHO African region, 2025. (**A**) Boxplots showing the distribution of turnaround times (days) from poliovirus isolation results to reporting of PCR-ITD results across laboratories. (**B**) Proportion of samples with final PCR-ITD results within 7 days of poliovirus isolation results. The dashed lines indicate the WHO-recommended targets: <7 days for turnaround time and >80% of samples meeting the timeliness threshold.

Figure 4A illustrates the distribution of PCR-ITD TAT for poliovirus isolates across the laboratories. Median TAT ranged from 0 to 7 days. South Africa showed the longest median TAT (median: 7 days; IQR: 6–7), followed by Ethiopia (median: 5 days; IQR: 2–8). Median TAT of 3 days were observed in Senegal (IQR: 1–4), Zambia (IOQ: 4–4), and Zimbabwe (IQR: 3–7). Most laboratories demonstrated relatively short PCR-ITD TATs, with median values between 0 and 2 days, including Algeria (IQR: 0–2), Madagascar (IQR: 0–3); Cote d’Ivoire (IQR: 0–1), and Cameroon CAR, Ghana, Ibadan (Nigeria), Kenya, DRC, and Ugandan, each reporting a median TAT of 2 days. Greater variability in PCR-ITD TAT was observed in Ethiopia and Zimbabwe, as reflected by their wider IQRs. Despite this, all laboratories except DRC met the WHO-recommended target of 80% of specimens receiving PCR-ITD results within 7 days of virus isolation (Figure 4B).

### 3.4. Timely Outbreak Notification Activating Rapid Response

**Figure 5:**
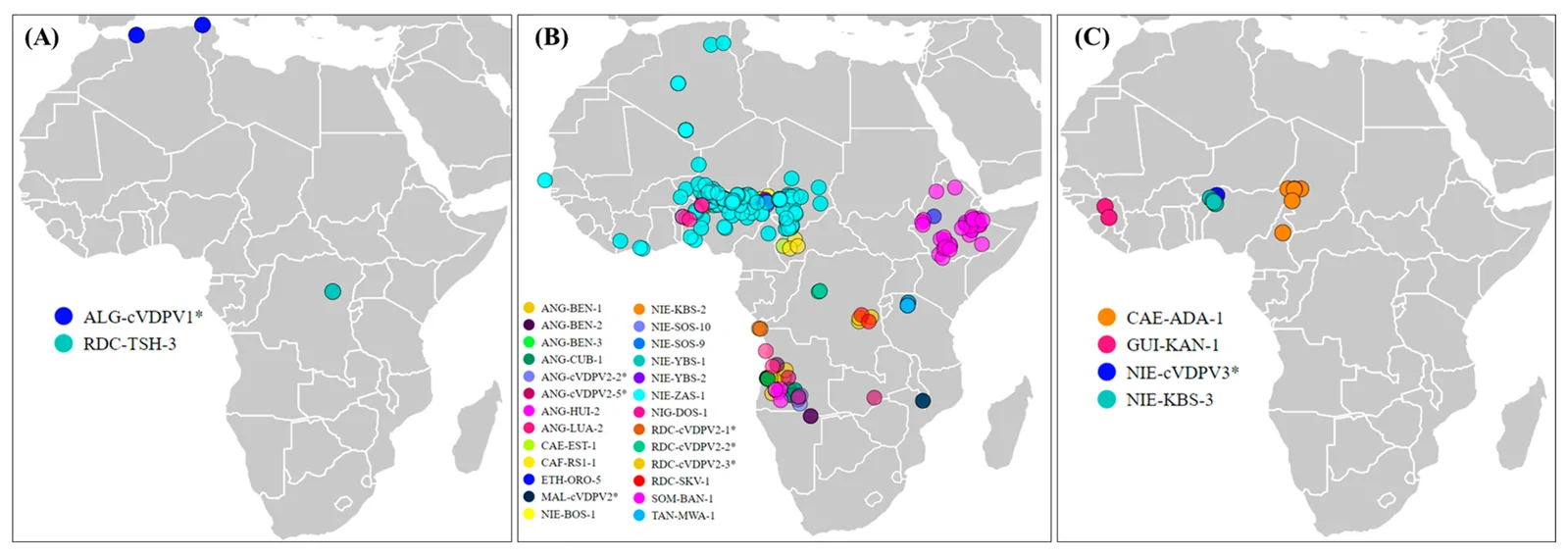
Geographic distribution of circulating Vaccine-derived Poliovirus (cVDPV) isolates by genetic lineage and serotype, WHO African region, 2025. Each point represents a single sequenced cVDPV isolate mapped to its geographical location, with colors denoting distinct genetic lineages based on sequence analysis. Asterisk (*) indicate isolates for which genetic lineage classification is pending. (**A**–**C**) Panels show the spatial distribution of cVDPV isolates by serotype: type 1 (**A**), type 2 (**B**), and type 3 (**C**). Some mapped isolates were associated with outbreaks confirmed prior to the 2025 study period but continued to circulate during the reporting period.

As shown in Table 1, between January and December 2025, a total of 22 distinct poliovirus outbreaks were reported across 10 countries in the WHO African region. The majority (19/22) were caused by cVDPV2, while two outbreaks were attributed to cVDPV3 and one outbreak to cVDPV1. Angola accounted for the highest number of distinct outbreak clusters, followed by Nigeria and Ethiopia, indicating sustained and repeated transmission in these settings. The nucleotide divergence from the Sabin vaccine strain varied widely across the outbreaks, ranging from 6 to 72 substitutions, reflecting heterogeneous evolutionary trajectories. Outbreaks attributed to cVDPV3 were limited to Cameroon, Chad, Nigeria, and Guinea, while a single cVDPV1 outbreaks was reported in Algeria. The number of viruses per outbreak ranged from, 2 to 12, with several, large transmission clusters observed in Nigeria and Angola (Figure 5), suggesting potentially prolonged transmission chains in these high-burden settings.

### 3.5. Poliovirus Sequencing, Timeliness, and Expansion Plans

In 2025, two new sequencing laboratories, one in Uganda and another at the University of Ibadan, Nigeria became operational, substantially improving the geographic distribution of sequencing capacity across the WHO African region (Figure 1B).

**Figure 6:**
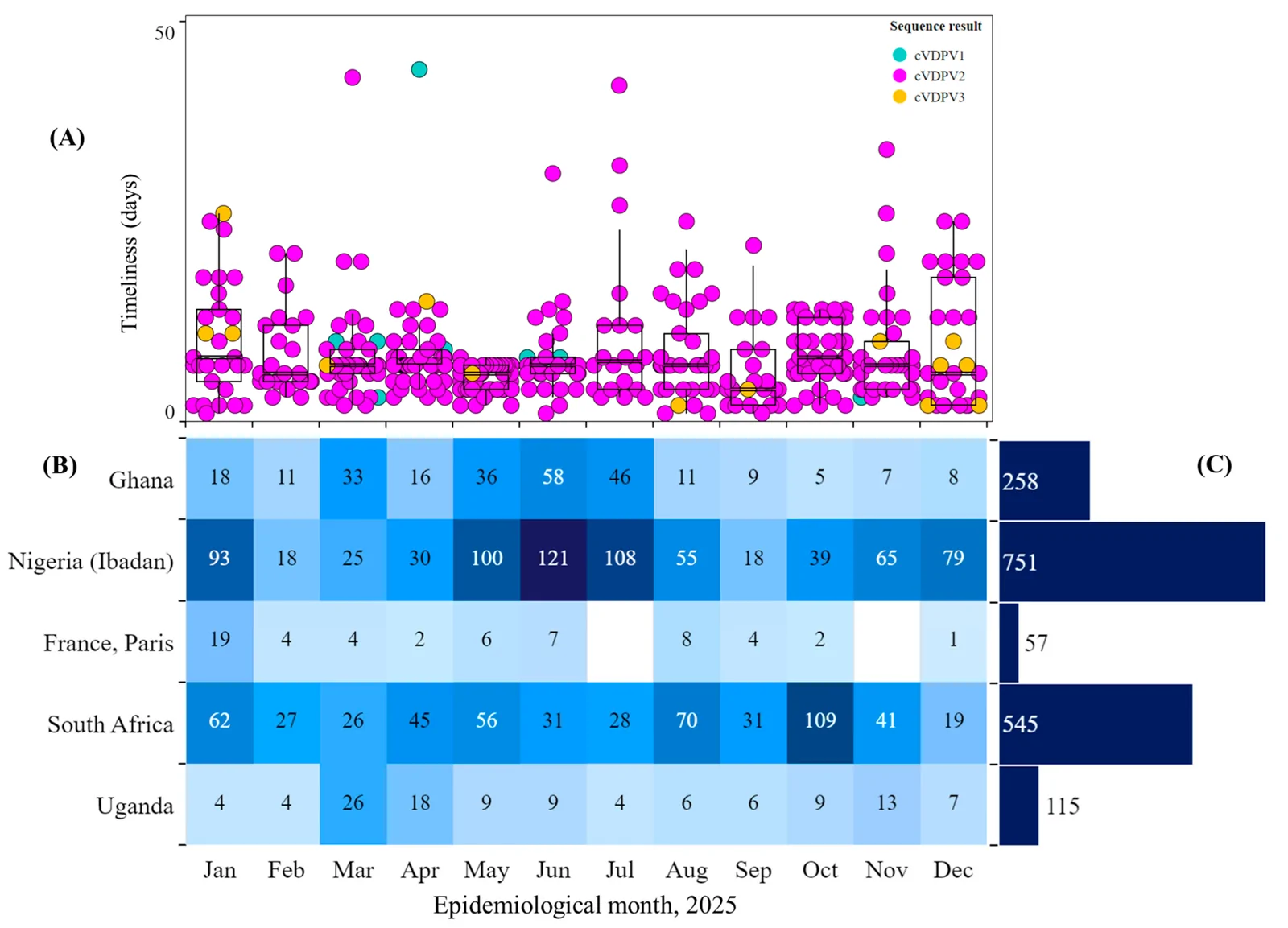
Timeliness and distribution of poliovirus sequencing by result type, time, and laboratory, WHO African region, 2025. (**A**) Distribution of sequencing timeliness (days from sample receipt to sequence result) by poliovirus sequencing outcome. Points represent individual isolates; boxplots indicate the median and interquartile range. (**B**) Number of poliovirus isolates sequenced by epidemiological month, showing temporal trends in sequencing activity. (**C**) Cumulative number of poliovirus isolates sequenced by laboratory, reflecting relative contribution and throughput across participating sequencing laboratories.

Figure 6C shows that a total of 1726 poliovirus samples were sequenced during the study period. Ibadan (Nigeria) polio laboratory processed the highest volume (*n* = 751), followed by South Africa polio laboratory (*n* = 545), Ghana polio laboratory (*n* = 258), Uganda polio laboratory (*n* = 115), and France polio laboratory (*n* = 57).

Overall, the regional TAT of sequencing results was 67%, indicating that approximately two-thirds of sequencing results were reported within the expected TAT target. Among the laboratories, Ghana and France showed the shortest TAT for 4 days (Figure 6A). Ghana showed more consistent performance (IQR: 4–6 days), while France had slightly greater variability (IQR: 3–7 days). Uganda also demonstrated relatively timely reporting, with a median of 5 days (IQR: 3–7). South Africa had a median TAT of 7 days with a narrow IQR (6–7), indicating stable and consistent performance. Conversely, Ibadan (Nigeria) showed the longest median TAT of 11 days and the widest variability (IQR: 8–18), suggesting potential operational challenges affecting sequencing timeliness.

The establishment of Ibadan (Nigeria) and Uganda sequencing polio laboratories has led to a marked reduction in sequencing delays for countries that previously depended on distant regional hubs. The most significant improvement was observed in countries, where isolates had traditionally been sent to the Centers of Disease Control and Prevention (CDC) laboratory in the United States. To further strengthen regional genomic surveillance and accelerate outbreak response, the WHO African region initiated a targeted expansion of sequencing laboratories in alignment with the GPSAP 2025–2026 (Table 2).

### 3.6. Laboratory Quality Assurance

#### 3.6.1. Annual Laboratory Accreditation Visits

**Figure 7:**
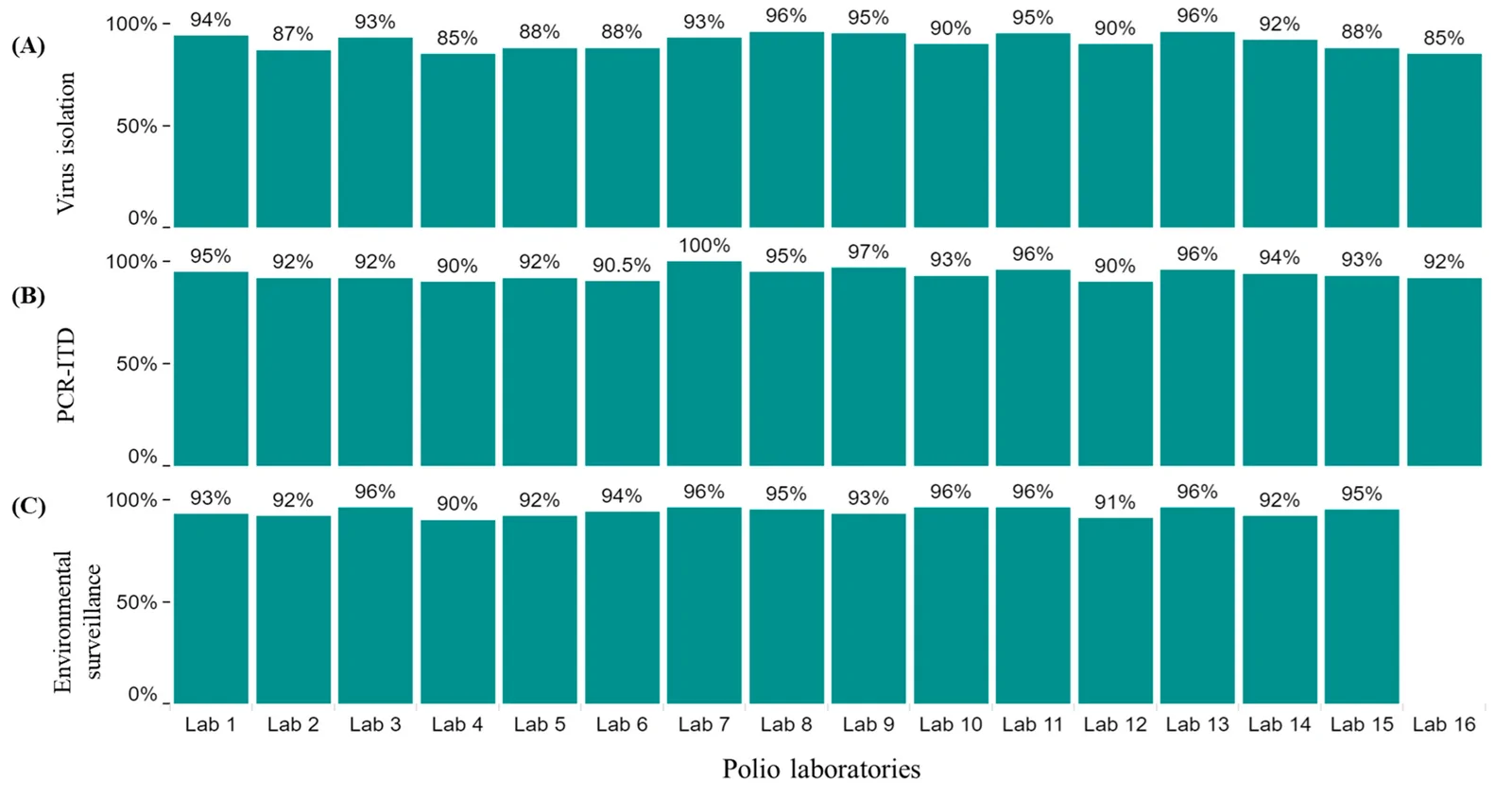
Performance outcomes of polio laboratory accreditation assessments and proficiency testing panels, WHO African region, 2024. (**A**) Virus isolation (target ≥ 80%), (**B**) PCR-ITD (target ≥ 90%), (**C**) Environmental surveillance (target ≥ 90%) (Note: environmental surveillance samples from Lab 16 were analyzed at the South Africa laboratory). For confidentiality and ethical considerations, laboratory identifiers have been coded and are not displayed by name in accreditation outputs, including tables and figures.

In 2024, the most recent year assessed, a total of 16 laboratories in the WHO African region underwent accreditation assessments. These evaluations, conducted using standardized GPLN checklists, focused on three technical domains; virus isolation (Figure 7A), PCR-ITD (Figure 7B), and environmental surveillance (Figure 7C). Laboratories in 7, 8, 9, 10, and 13 demonstrated consistently high performance across all domains. However, laboratories 2 and 4 achieved adequate scores in PCR-ITD and environmental surveillance but performed slightly lower in virus isolation. Two laboratories were evaluated exclusively for environmental surveillance and both were marked as “Passed,” while Laboratory 16 received provisional accreditation due to incomplete data during review period.

Accreditation missions were carried out by multidisciplinary teams comprising experts from WHO African Regional Office (WHO AFRO), Inter-Country Support Teams (ISTs), and technical staff from peer laboratories in the region, with exclusion of personnel from the laboratory under review to avoid conflicts of interest. Beyond compliance with quality standards, these visits also facilitated peer learning, enabling laboratories to share best practices, troubleshoot common challenges, and strengthen regional collaboration. Complementary supervisory missions were also conducted to provide hands-on support, monitor the implementation of routine activities; pilot new sequencing approaches, and reinforce data management practices. By the end of 2024, all polio laboratories in the region were accredited both AFP and environmental surveillance sample processing, underscoring their readiness and sustained contribution to the regional eradication efforts.

#### 3.6.2. Trainings and Capacity Building of the Workforce

As part of ongoing efforts to strengthen laboratory quality assurance, multiple rounds of targeted training were conducted across the polio laboratory network to enhance technical capacity and ensure adherence to standardized procedures. In 2024, more than 190 laboratory staff members including technicians and supervisors from all accredited polio laboratories participated in a one-week training session.

The sessions combined theoretical instruction with practical modules on virus isolation, cell culture techniques, environmental surveillance, and genomic sequencing, using standardized WHO training materials. Hands-on components were delivered in collaboration with the RRLs at the National Institute for Communicable Diseases (NICD) in South Africa, ensuring high-quality instruction grounded in real laboratory practice.

## 4. Discussion

Our study highlights the critical role of the African polio laboratory network in supporting poliovirus surveillance and eradication efforts across diverse and resource-constrained settings. Building on these findings, the discussion explores laboratory performance, achievements, and challenges in ensuring timely and reliable detection of polioviruses.

Workload distribution analysis revealed major disparities across the network. Laboratories in Nigeria and DRC processed more than half of all AFP stool specimens, reflecting their population size and higher AFP case burden. While expected, this disproportionate workload raises concerns about capacity strain and risks of delays, a challenge similarly documented in other disease laboratory networks such as tuberculosis, HIV [28,29]. Addressing this requires sustained investment in human resources, logistics, and where feasible, mechanisms for workload redistribution.

Laboratory performances in virus isolation and PCR-ITD, two primary functions of early poliovirus detection, were generally strong, with most laboratories meeting WHO-recommended targets for timeliness [30]. However, delays were noted in Laboratory 6 and 15, particularly virus isolation. These delays were largely attributed to logistics, ongoing renovation activities, and supply chain constraints, which can compose timely outbreak confirmation and response in high-priority countries.

The outbreak notification procedures of the African polio laboratory network, aligned with the GPEI outbreak response guideline [31], remain central to ensuring rapid and coordinated response. Laboratory confirmation triggers immediate notification to national and regional coordination structures, activation of emergency operations centers within 72 h, rapid risk assessment, and preparation of response teams (Figure 7). This integration of laboratory evidence into operational decision-making is essential for interrupting transmission and accelerating eradication, particularly in high-risk and cross-border transmission settings [2,14,32].

The observed predominance of cVDPV2 among AFP associated detections highlights the need for continued genomic surveillance and prospective investigations to better understand the evolutionary dynamics and biological characteristics of circulating strains [33,34,35]. Although cVDPV2 emergence is primarily associated with prolonged replication and evolution of attenuated Sabin-derived viruses in under-immunized populations, additional factors such as immunodeficiency-associated prolonged infections and specific genetic changes potentially affecting neurovirulence may contribute to viral fitness and pathogenicity. Future studies integrating detailed genomic analyses and host immune assessments are needed to further characterize these factors and their implications for poliovirus eradication and post-eradication surveillance.

Our analysis demonstrates that the establishment of new sequencing laboratories has markedly reduced turnaround times for genomic analysis. Countries that previously relied on distant regional or global hubs now benefit from more rapid outbreak confirmation and activation of outbreak response strategies. These findings reinforce the value of regional approaches to decentralized genomic capacity, showing that alignment with international guidelines can shorten the detection-to-response timeline and enhance eradication efforts [23].

Beyond expanding geographic sequencing capacity, the African polio laboratory network has begun integrating innovative technologies, including portable platforms such as MinION (Oxford Nanopore technology) and protocols for Direct Detection and Nanopore Sequencing (DDNS) [36]. These tools enable rapid genomic analysis by avoiding traditional virus isolation, significantly reducing the interval from specimen collection to confirmation of poliovirus transmission. This aligns with evidence from other studies demonstrating that the portable sequencing technologies, particularly MinIon, can accurately identify and characterize viral and bacterial pathogens while strengthening outbreak response and disease eradication efforts [37,38,39,40,41]. As these technologies are scaled across the network, they are expected to enhance epidemiology, molecular surveillance, and real-time public health intelligence.

Quality assurance was a core operational component of the African polio laboratory network, consistent with other regional networks, such as the Spanish national polio laboratory system [42]. The network maintained robust performance through its quality assurance protocols, which included regular accreditation and external PT panels. All laboratories achieved successful accreditation for virus isolation and ITD and environmental surveillance. Laboratory visits were further strengthened by peer-to-peer learning and supervisory missions [2,5,14]. The majority of laboratories successfully completed proficiency testing panels, demonstrating high technical competency and operational readiness [2,5,14]. These measures enabled the network to sustain WHO standards despite internal and external challenges. Lessons from measles and rubella laboratory networks similarly highlight the value of routine PT and accreditation in maintaining diagnostic quality [2,43,44,45]. Additionally, training remains a key element of quality improvement, equipping personnel with the skills necessary for accurate and timely virus detection, which is critical for guiding outbreak response and eradication strategies.

The African polio laboratory network also supports countries in the WHO Eastern Mediterranean Region (EMRO), reflecting strong cross-regional collaboration. These partnerships facilitate information exchange and joint outbreak analysis, particularly in the Horn of Africa, where cross border virus importation is common [46]. Such synergy improves operational efficiency, enables timely decision-making, and ensures that surveillance and response strategies are coordinated across regions, contributing to a unified eradication approach [46].

A major operational challenge reported by the laboratories was shortages of critical reagents and supplies, which disrupted sample processing and delayed reporting of results [2]. The network mitigated these issues by reallocating reagents from laboratories with surplus stocks to those in need. This collaborative mechanism facilitated continued testing operations and demonstrated strong inter-laboratory coordination. Similar challenges have been reported in previous studies [2,14,47] and in other disease eradication programs, such as measles and yellow fever, where procurement and logistics bottlenecks delayed diagnostics and hampered outbreak response despite robust surveillance systems [2,14]. Systematic solutions are necessary to safeguard polio eradication progress and extend the benefits of laboratory surveillance to other epidemic-prone diseases.

While the study provides valuable insights, limitations must be acknowledged. Data were exclusively derived from AFP stool specimens, a gold standard and principal method for polio eradication program [48,49], which may underrepresent the full operational workload of the laboratories. Furthermore, findings on sequencing performance reflect the available samples at the time of data extraction; further data may refine these trends.

## 5. Conclusions

This study highlights the contributions of the African polio laboratory network in timely poliovirus detection, outbreak response, and genomic surveillance across the WHO African region. By expanding sequencing capacity, achieving strong accreditation outcomes, and implementing collaborative mechanisms to mitigate supply chain challenges, the laboratory network has demonstrated its readiness to support the final stages of polio eradication in the region.

Despite these achievements, performance disparities and supply chain constraints persist. Sustained investment in infrastructure, logistics, and technical oversight is essential to ensure that all laboratories consistently deliver high-quality, timely results. The adoption of innovative sequencing technologies, such as MinION and DDNS, further strengthens near real-time molecular epidemiology, accelerating outbreak response and supporting broader preparedness objectives. As eradication goals approach, policymakers and global health stakeholders should prioritize integrating this network into regional health security frameworks, ensuring its continued contribution to the detection and response to emerging public health threats.

## Figures and Tables

**Table 1:** Characteristics of circulating Vaccine-derived Poliovirus (cVDPV) new outbreaks reported in the WHO African region, 2025.

Outbreak Code	Outbreak Notification Date	Country	Serotype	Emergence Group	Index Virus	Most Recent Virus	Nuclotide Change from Vaccine Strain (Min–Max)	Total Number of Viruses
DZA-ETF-CVDPV1ALG-CVDPV1-25	15/09/2025	ALGERIA	Type 1	ALG-cVDPV1 *	25/01/2025	17/03/2025	46–72	2
AGO-BEN-ANGBEN1-CVDPV2-24	17/03/2025	ANGOLA	Type 2	ANG-BEN-1	12/12/2024	06/07/2025	10–17	8
AGO-BEN-ANGBEN2-CVDPV2-25	24/03/2025	ANGOLA	Type 2	ANG-BEN-2	06/01/2025	03/10/2025	6–12	5
AGO-BEN-ANGBEN3-CVDPV2-24	07/04/2025	ANGOLA	Type 2	ANG-BEN-3	24/11/2024	11/08/2025	12–14	5
AGO-HUI-CVDPV2ANG-CVDPV2-25	22/09/2025	ANGOLA	Type 2	ANG-BEN-3	23/06/2025	11/08/2025	15–16	5
AGO-CUB-ANGCVDPV22-CVDPV2-25	13/10/2025	ANGOLA	Type 2	ANG-CUB-2	16/08/2025	27/10/2025	13–23	4
AGO-CUB-ANGCVDPV23-CVDPV2-25	27/10/2025	ANGOLA	Type 2	ANG-CUB-1	31/08/2025	13/10/2025	12–16	4
AGO-LUA-ANGCVDPV24-CVDPV2-25	27/10/2025	ANGOLA	Type 2	ANG-LUA-2	26/06/2025	10/09/2025	12–13	2
AGO-CUA-ANGCVDPV25-CVDPV2-25	24/11/2025	ANGOLA	Type 2	ANG-BEN-2	04/09/2025	03/10/2025	9–14	5
CMR-ADA-CVDPV3-CVDPV3-25	21/07/2025	CAMEROON	Type 3	CAE-ADA-1	30/05/2025	30/05/2025	52–52	2
CAF-RS2-CAEEST1-CVDPV2-25	05/05/2025	CENTRAL AFRICAN REPUBLIC	Type 2	CAE-EST-1	25/03/2025	25/03/2025	6–12	1
CAF-RS1-CVDPV2CAF-CVDPV2-25	18/08/2025	CENTRAL AFRICAN REPUBLIC	Type 2	CAF-RS1-1	26/04/2025	07/06/2026	10–15	7
TCD-NDJ-CHACVDPV2-CVDPV2-25	31/03/2025	CHAD	Type 2	CAE-ADA-1	30/05/2025	30/05/2025		1
TCD-CHB-CVDPV3-CVDPV3-25	25/08/2025	CHAD	Type 3	CAE-ADA-1	20/06/2025	18/10/2025	43–60	5
COD-KCT-CVDPV2RDC-CVDPV2-25	07/07/2025	DEMOCRATIC REPUBLIC OF THE CONGO	Type 2	RDC-KCT-1	16/05/2025	16/05/2025	11–11	2
ETH-ORO-ETHORO5-CVDPV2-24	14/04/2025	ETHIOPIA	Type 2	ETH-ORO-5	01/12/2024	27/02/2025	19–20	3
ETH-SOM-CVDPV2ETH-CVDPV2-24	23/06/2025	ETHIOPIA	Type 2	ETH-SOM-2	23/09/2024	23/09/2024	16–16	1
NAM-KAV-ANGBEN2-CVDPV2-25	24/11/2025	NAMIBIA	Type 2	ANG-BEN-2	14/10/2025	04/03/2026	8–17	7
NGA-YBS-NIEYBS2-CVDPV2-25	24/03/2025	NIGERIA	Type 2	NIE-YBS-2	06/01/2025	03/10/2025	15–42	5
NGA-SOS-CVDPV2NIE-CVDPV2-25	08/09/2025	NIGERIA	Type 2	NIE-SOS-9	23/05/2025	27/07/2025	8–13	3
NGA-SOK-NIECVDPV23-CVDPV2-25	27/10/2025	NIGERIA	Type 2	NIE-SOS-10	30/06/2025	27/03/2026	13–19	7
TZA-MWA-TANMWA1-CVDPV2-24	10/03/2025	TANZANIA	Type 2	TAN-MWA-1	18/11/2024	16/12/2025	15–32	9

* The cVDPV emergence group has not yet been determined.

**Table 2:** Phased expansion of poliovirus sequencing laboratories in the WHO African region and sequencing techniques applied, 2025.

S/N	Polio Laboratory Name	Country	Category	Sequencing Technique
1	Institute Pasteur d’Algérie	Algeria	Phase 2	Sanger
2	Centre Pasteur du Cameroun	Cameroon	Phase 1	MinIon
3	Institute Pasteur de Cote d’Ivoire	Cote d’Ivoire	Phase 3	MinIon
4	Institute Pasteur de Bangui	CAR	Phase 3	MinIon
5	Institute National de Recherche Biomédicale	DRC	Phase 1	Both MinIon and Sanger
6	Ethiopian Public Health Institute	Ethiopia	Phase 3	Sanger
7	Kenya Medical Research Institute	Kenya	Phase 1	Both MinIon and Sanger
8	Institute Pasteur de Madagascar	Madagascar	Phase 2	Sanger
9	University of Maiduguri Teaching Hospital Polio Laboratory	Nigeria	Phase 2	Sanger
10	Institute Pasteur de Dakar	Senegal	Phase 1	MinIon
11	University Teaching Hospital Virology Laboratory	Zambia	Phase 3	MinIon
12	National Microbiology Reference Laboratory	Zimbabwe	Phase 3	MinIon

## Data Availability

The data used for this study is available with justifiable request.

## Abbreviations

The following abbreviations are used in this manuscript:

AFP	Acute Flaccid Paralysis
DDNS	Direct Detection and Nanopore Sequencing
GPEI	Global Polio Eradication Initiative
GPLN	Global Polio Laboratory Network
ITD	Intratypic Differentiation
IQR	Interquartile Range
PCR	Polymerase Chain Reaction
TAT	Turnaround Time
